# An analysis of the imaging features and diagnostic value of osteoid osteoma on DR, CT, and MRI

**DOI:** 10.3389/fmed.2026.1890873

**Published:** 2026-08-05

**Authors:** Xian Jun He, Yi Chen Jiang, Peng Yang, Li Ling Lu, Chun Xin Huang, Chun Yan Li

**Affiliations:** 1 Department of Radiology, The First Affiliated Hospital of Guangxi Medical University, Nanning, China; 2 Department of Radiology, Minzu Hospital of Guangxi Zhuang Autonom, Nanning, China

**Keywords:** Osteoid Osteoma, DR, CT, MRI, Diagnostic Value

## Abstract

**Objective:**

To compare the diagnostic value of digital radiography (DR), computed tomography (CT), and magnetic resonance imaging (MRI) in osteoid osteoma (OO).

**Materials and methods:**

A retrospective analysis was performed on imaging data of 99 patients with pathologically confirmed OO (84 underwent DR, 86 underwent CT, and 36 underwent MRI). Detection rates of lesion features and diagnostic concordance rates for DR, CT, and MRI were calculated; intergroup comparisons were conducted using Fisher’s exact test.

**Results:**

Among 99 OO patients, 74.7% were male (mean age, 15.41 ± 9.98 years). The proximal femur was the most common site (42.4%). For imaging features, CT showed detection rates of 100, 91.9, and 89.5% for the nidus, bone sclerosis, and intranidal calcification, respectively all higher than those of DR (52.4, 69.0, 16.7%) and MRI (44.4, 58.3, 55.6%). MRI achieved an 83.3% detection rate for perilesional bone marrow edema. All 23 intra-articular OOs were accompanied by joint effusion and synovitis. Diagnostic concordance rate comparison revealed that CT’s overall rate was significantly higher than that of DR and MRI (*p* < 0.001).

**Conclusion:**

CT provides the best visualization of the OO nidus, surrounding bone sclerosis, and intranidal calcifications, with the highest diagnostic accuracy, making it the optimal imaging modality. MRI has a distinct advantage in edema detection, aiding in the assessment of intra-articular lesion activity.

## 1 Introduction

Osteoid osteoma (OO) is a common benign osteogenic tumour, accounting for 3% of all bone tumours and approximately 10–12% of biopsy-confirmed benign bone tumours (1, 2). The condition primarily affects boys and young men aged 7–25 years; 70% of cases occur before the age of 20, and it is rare in children under 5 or in individuals over 35 (3–5). Typical clinical presentations include nocturnal pain, which responds well to non-steroidal anti-inflammatory drugs (NSAIDs) (6–9). OOs commonly affect the long bones of the lower limbs, but may occur in any bone, and sometimes present with atypical clinical and radiological features (4, 10). Early and accurate identification is crucial for selecting appropriate treatment modalities and avoiding unnecessary invasive procedures or prolonged delays in treatment.

DR, CT, and MRI are widely used in clinical practice to evaluate suspected OOs. but the differences among these imaging modalities in terms of diagnostic accuracy remain unclear. Existing studies have largely focused on describing the overall imaging characteristics of OOs (11–14). For example, Yazol et al. (11) reported that typical OO on MRI presents as a central hypointense lesion with marked perilesional bone marrow and soft tissue edema. Bosanac et al. (12) noted that hip OO often manifests as focal femoral neck/acetabular sclerosis with a nidus, presenting with unexplained pain (easily misdiagnosed as synovitis, stress fracture, or early osteonecrosis) and prominent peripheral bone marrow edema on MRI, though nidi may be atypical. Bedoya et al. (13) found that intra-articular OO may show normal or subtle sclerotic changes on X-ray, with synovitis, joint effusion, and marrow edema-like signals on MRI; CT is the gold standard for nidus detection. Few studies have systematically compared the differences in diagnostic agreement rates between DR, CT, and MRI for OOs within the same cohort, and the differences in detection rates of specific imaging features among these imaging modalities have not yet been fully elucidated.

Therefore, this study aims to objectively evaluate the imaging characteristics and diagnostic concordance rates of DR, CT, and MRI in OOs, thereby providing evidence-based guidance for the selection of imaging modalities and diagnostic workflows in clinical practice.

## 2 Materials and methods

### 2.1 Patients

**Figure 1:**
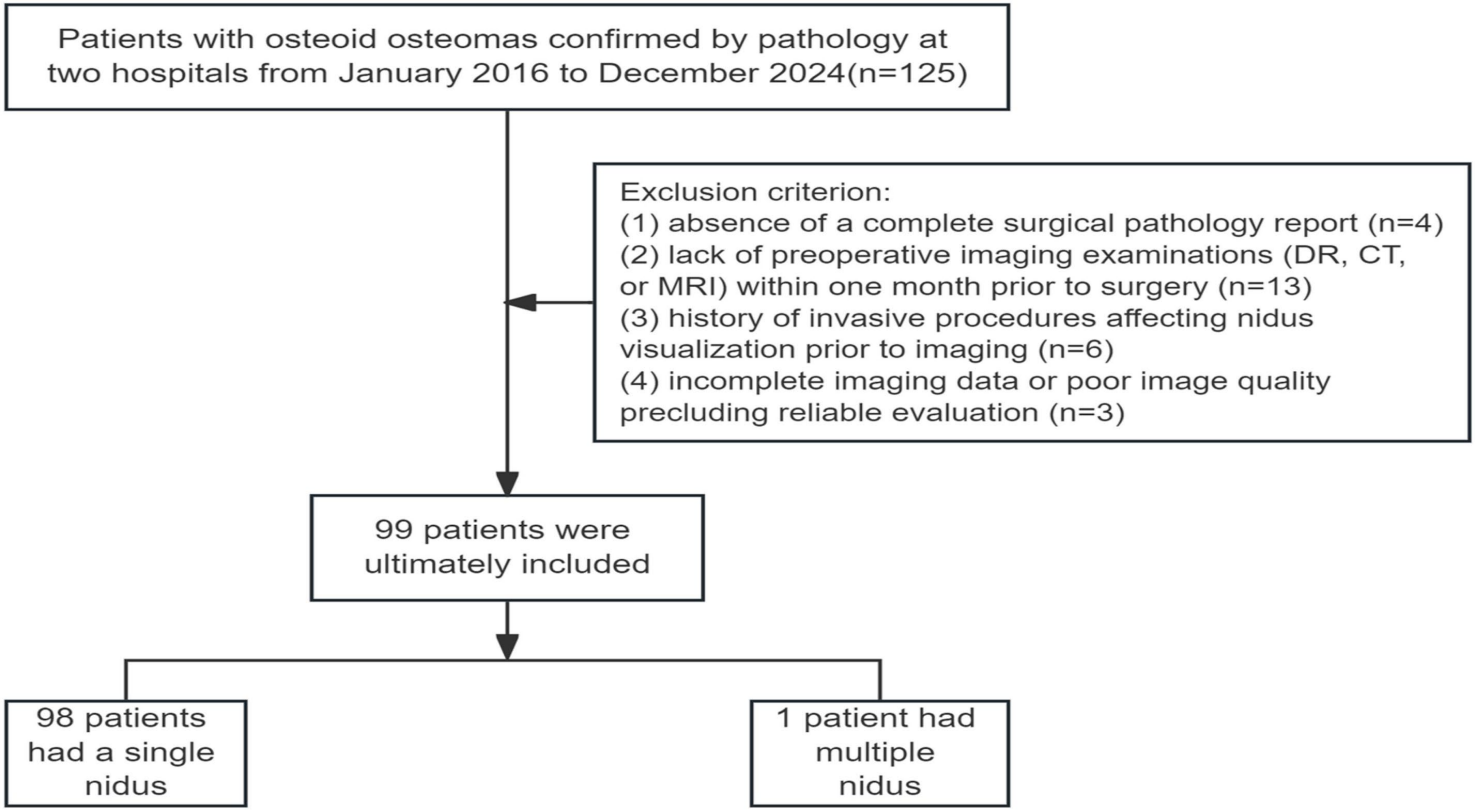
Study flow chart.

Clinical and imaging data were retrospectively collected from patients with histopathologically confirmed OO at two hospitals between January 2016 and December 2024. Exclusion criterion: (1) absence of a complete surgical pathology report; (2) lack of preoperative imaging examinations (DR, CT, or MRI) within 1 month prior to surgery; (3) history of invasive procedures affecting nidus visualization prior to imaging; (4) incomplete imaging data or poor image quality precluding reliable evaluation. A flow diagram illustrating the patient selection process is provided in Figure 1. Patients with a radiological diagnosis suggestive of OO but without pathological confirmation were excluded. A total of 99 patients were ultimately included, including 98 with a single nidus and 1 with multiple nidus (two distinct nidus within the same femoral shaft, both covered in a single imaging field). Of the 99 patients, 84 underwent DR, 86 underwent CT, 36 underwent MRI, and 10 underwent MRI with contrast enhancement. 26 underwent all three imaging modalities (DR, CT, and MRI) simultaneously.

### 2.2 Imaging techniques

DR examinations were performed using Philips Digital Diagnost (Netherlands), Siemens YSIO (Germany), Siemens Axiom Aritos VX (Germany), and SAMSUNG GC85A (Korea). CT scans were acquired on Siemens SOMATOM Force, Somatom Sensation 64, Somatom Sensation 128, and SOMATOM Definition Edge scanners (all Germany). CT scans are always performed using a tube voltage of 120 kV. MRI examinations were performed on 1.5 T or 3.0 T systems including Philips Achieva/Intera 1.5 T (Netherlands), GE Signa 1.5 T and SIGNA Premier 3.0 T (USA), Siemens MAGNETOM Verio 3.0 T (Germany), and Canon Vantage ELAN 1.5 T (Japan). Standard scanning sequence: axial T1-weighted images (T1WI), axial and coronal T2-weighted images (T2WI), and coronal STIR sequences. For contrast-enhanced scanning, gadolinium diethylenetriamine pentaacetic acid (Gd-DTPA) is administered via the antecubital vein (at a dose of 0.1 mmol/kg) prior to the T1WI scan, with an infusion rate of 1 mL/s.

### 2.3 Imaging analysis

Two radiologists with 7 and 12 years of experience in musculoskeletal imaging (XH and PY) independently reviewed all imaging studies without conferring with each other or accessing the original clinical reports, and provided probable diagnoses. The diagnostic value of imaging examinations is evaluated using the rate of imaging-suggestive diagnoses, the rate of definitive diagnoses, and the overall diagnostic concordance rate. The overall diagnostic concordance rate is the sum of the rate of imaging-suggestive diagnoses and the rate of definitive diagnoses. Discrepancies between the two radiologists were resolved by consensus. Cohen’s kappa coefficient was used to assess inter-observer agreement. The criteria for interpreting agreement were as follows: <0.20, poor; 0.21–0.40, fair; 0.41–0.60, moderate; 0.61–0.80, good; and 0.81–1.00, excellent.

#### 2.3.1 OO classification

**Figure 2:**
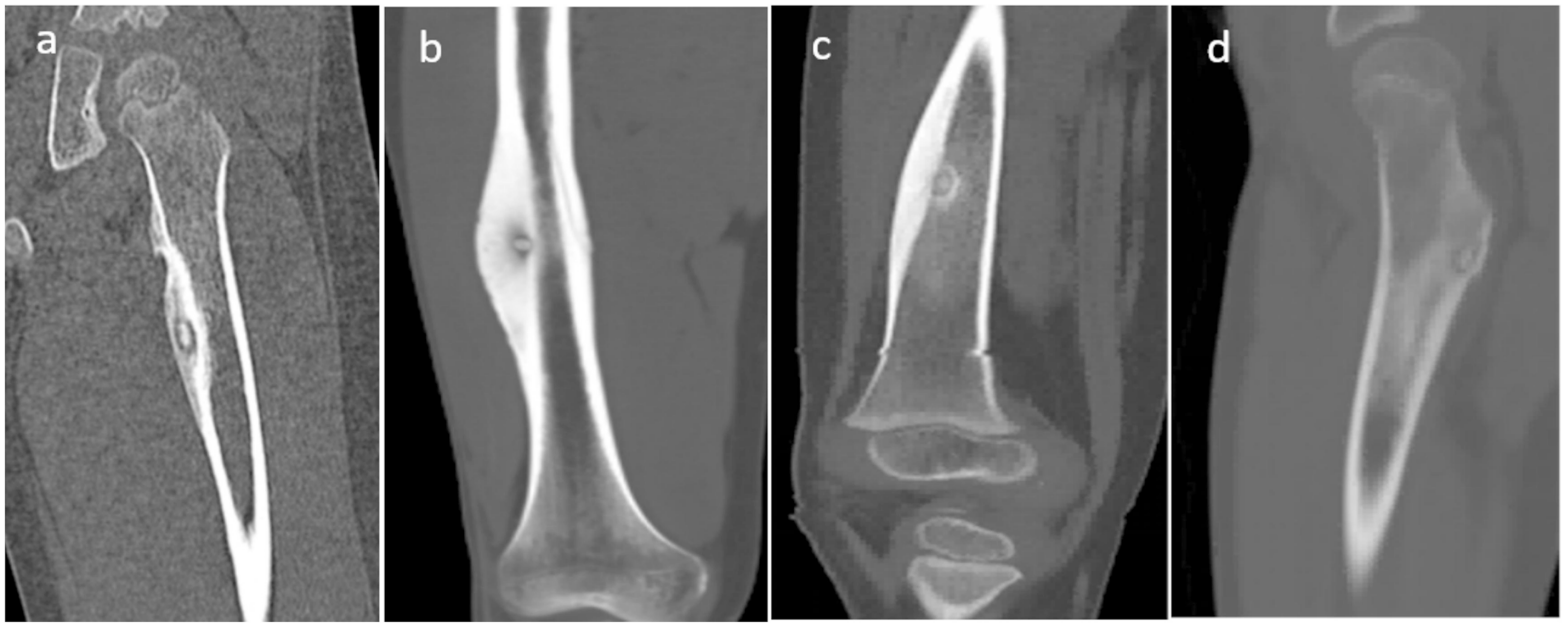
Classification of OO. **(a,b)** Cortical type; **(c,d)** Endosteal type.

Cortical type: Defined as nidus located partially or entirely within the cortical bone. Endosteal type: Defined as nidus located within the cancellous bone adjacent to the cortical bone. The classification of OO is shown in Figure 2.

#### 2.3.2 Identification of imaging features

**Figure 3:**
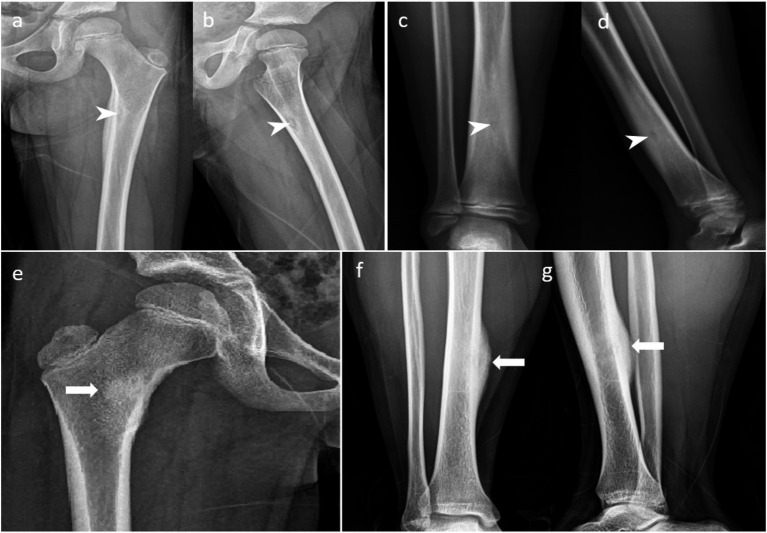
Schematic diagram of DR findings. Nidus (swallowtail triangular arrows in **a–d**), bone sclerosis (thick arrows in **e–g**).

DR: Nidus (present/absent, defined as round or oval areas of low-density lucency); Bone sclerosis (present/absent, manifested as cortical thickening and increased bone density). Schematic diagram of DR signs is shown in Figure 3.

**Figure 4:**
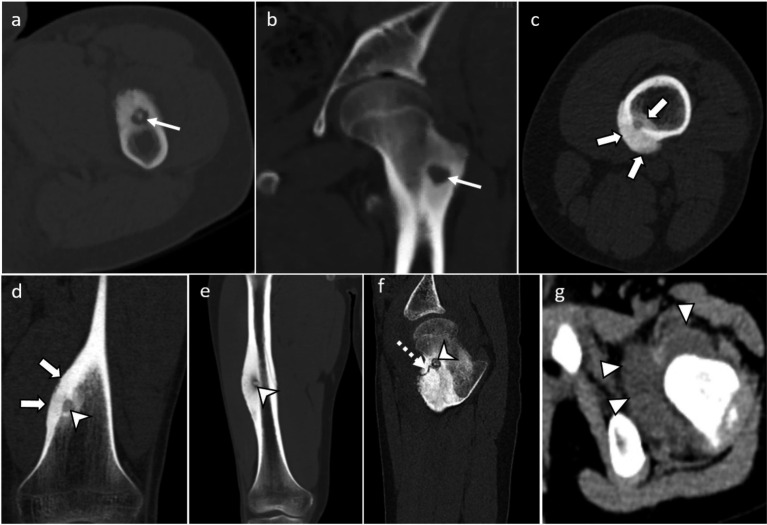
Schematic diagram of CT findings: Nidus (thin arrows in **a,b**), bone sclerosis (thick arrows in **c,d**), calcification within the nidus (swallowtail triangular arrows in **d–f**), vascular groove sign (dashed arrow in **f**), and surrounding soft tissue edema (triangular arrow in **g**).

CT: Nidus (present/absent, defined as round or oval hypodense areas). Bone sclerosis (present/absent, manifested as cortical thickening and increased bone density). Calcification within the nidus (present/absent, defined as a CT value greater than 100 HU). Vascular groove sign (present/absent, defined as a radiolucent linear or serpentine groove extending downwards from the surface of the cortical bone and periosteum to or near the radiolucent lesion). Surrounding soft tissue edema (present/absent; primarily manifested as localized soft tissue and localized density changes, with indistinct borders). Schematic diagram of CT signs is shown in Figure 4.

**Figure 5:**
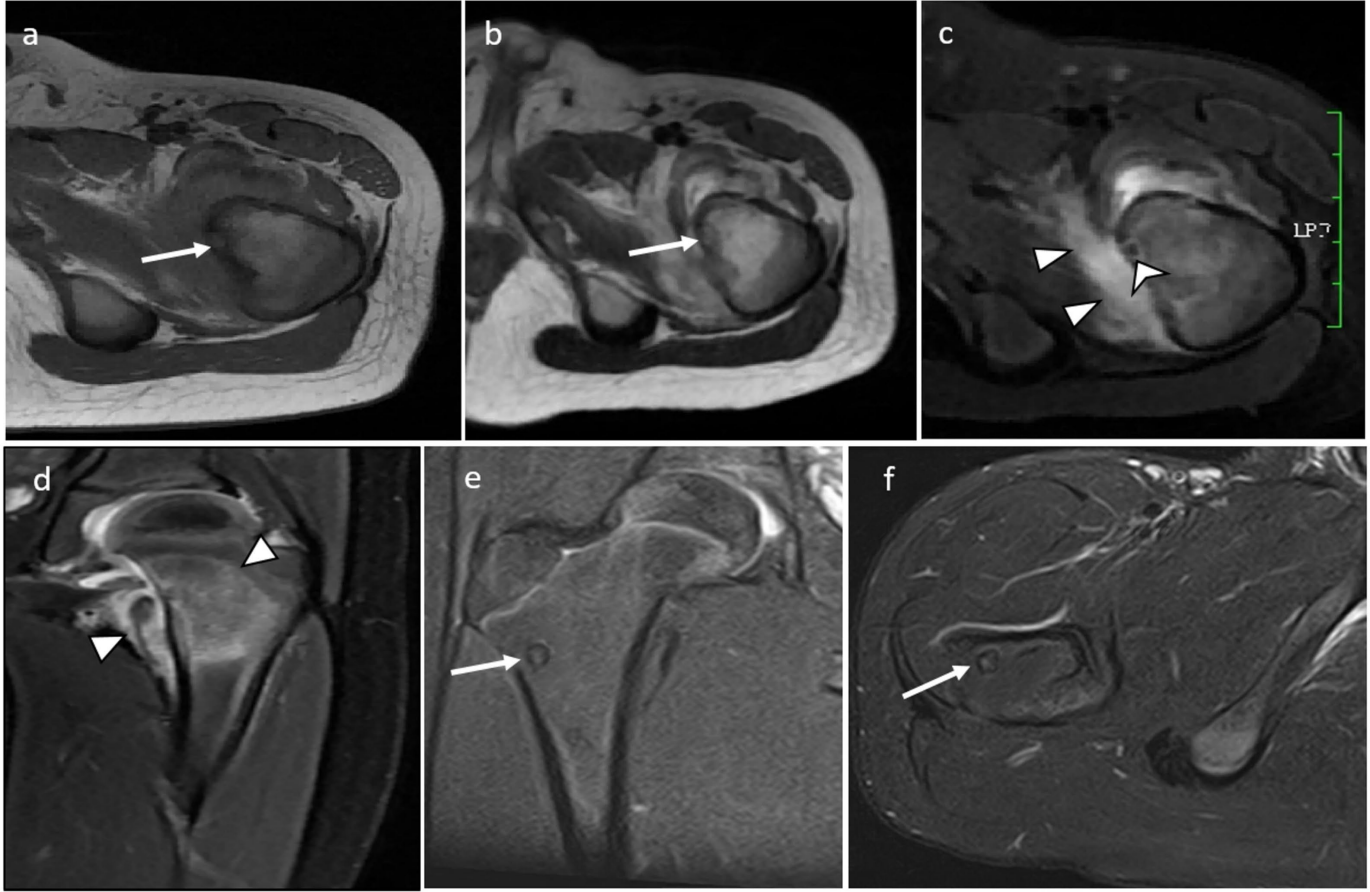
Schematic diagram of MRI findings. **(a–d)** The nidus in the left femoral neck appears as a slightly hyperintense signal on T1WI and T2WI (small arrow); calcification within the nidus (triangular arrow); and oedema of the bone marrow and surrounding soft tissues appears as a hyperintense signal (triangle). **(e,f)** An isolated nidus in the right trochanter of the femur, with no obvious bone marrow or soft tissue edema (thin arrow).

MRI: Nidus (absent, equivocal, or definite; defined as a slightly hyperintense signal on both T2-weighted and T1-weighted images, with calcifications within the focus appearing hypointense). Sclerotic signal changes (present/absent; manifested as a hypointense signal around the lesion on T1-weighted and T2-weighted images). Bone marrow oedema (present/absent; defined as ill-defined areas of increased signal intensity on fat-suppressed T2-weighted or STIR sequences within the bone marrow adjacent to the nidus). Surrounding soft tissue oedema (present/absent; defined as increased signal intensity on T2-weighted or STIR sequences in the peri-lesional soft tissues, with indistinct borders). Schematic diagram of MRI signs is shown in Figure 5.

#### 2.3.3 Signs of intra-articular OO (joint effusion and synovitis)

**Figure 6:**
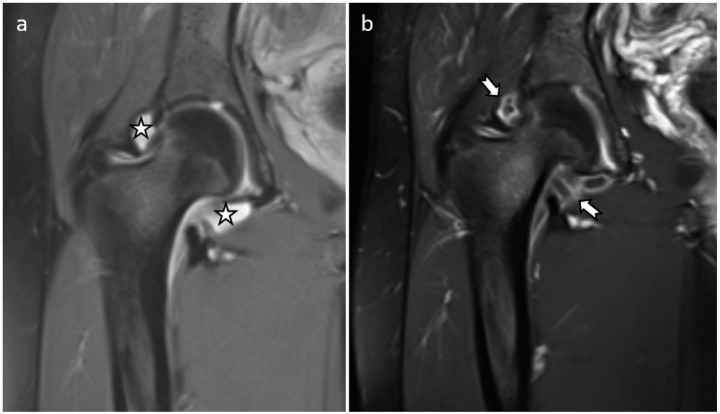
Joint effusion and synovitis. **(a)** T2-weighted image with fat suppression; **(b)** T1-weighted image with fat suppression and contrast enhancement. Marked effusion within the right hip joint cavity (asterisk), and marked thickening and enhancement of the synovium (tail-of-swallowtail arrow).

Intra-articular OO was defined as a nidus located within the joint capsule or immediately adjacent to the articular surface, with imaging evidence of joint involvement (effusion and/or synovitis). Joint effusion is defined as a condition in which the joint capsule is filled with fluid; on CT, it appears as an area of water-like density, and on MRI, it appears as a high signal on T2-weighted images (T2WI) and a low signal on T1-weighted images (T1WI), with a depth greater than 2 mm. Synovitis is defined as marked thickening of the synovial membrane (≥2 mm) with marked enhancement following contrast administration. Joint effusion and synovitis is shown in Figure 6.

#### 2.3.4 Imaging diagnostic findings

DR: Cannot be diagnosed (no nidus, no sclerosis); Suggestive (sclerosis within the bone cortex or the medullary cavity adjacent to the bone cortex; sclerosis and low density; low density); Definitive (nidus; nidus with sclerosis).

MRI: Cannot be diagnosed (no nidus, no sclerosis signals), suggestive (thickening of the cortical bone or sclerosis signals, with or without medullary oedema; nidus of uncertain appearance), definitive (nidus, nidus with sclerosis signals).

CT: Presents as a round or oval hypodense nidus; nidus can be confirmed even in the absence of calcification (based on sclerosis, location, morphology, and surrounding soft tissue oedema).

## 3 Statistical analysis

Statistical analysis was performed using SPSS 26.0 software. Continuous variables were presented as mean ± standard deviation (*x̄* ± *s*); categorical variables as frequencies (percentages, *n*/%). Rates of definitive diagnosis, suggestive diagnosis, and overall diagnostic concordance were calculated and compared among groups. Intergroup comparisons were performed using Fisher’s exact test. A *p*-value < 0.05 was considered statistically significant.

## 4 Results

### 4.1 Age, sex and site of origin

**Figure 7:**
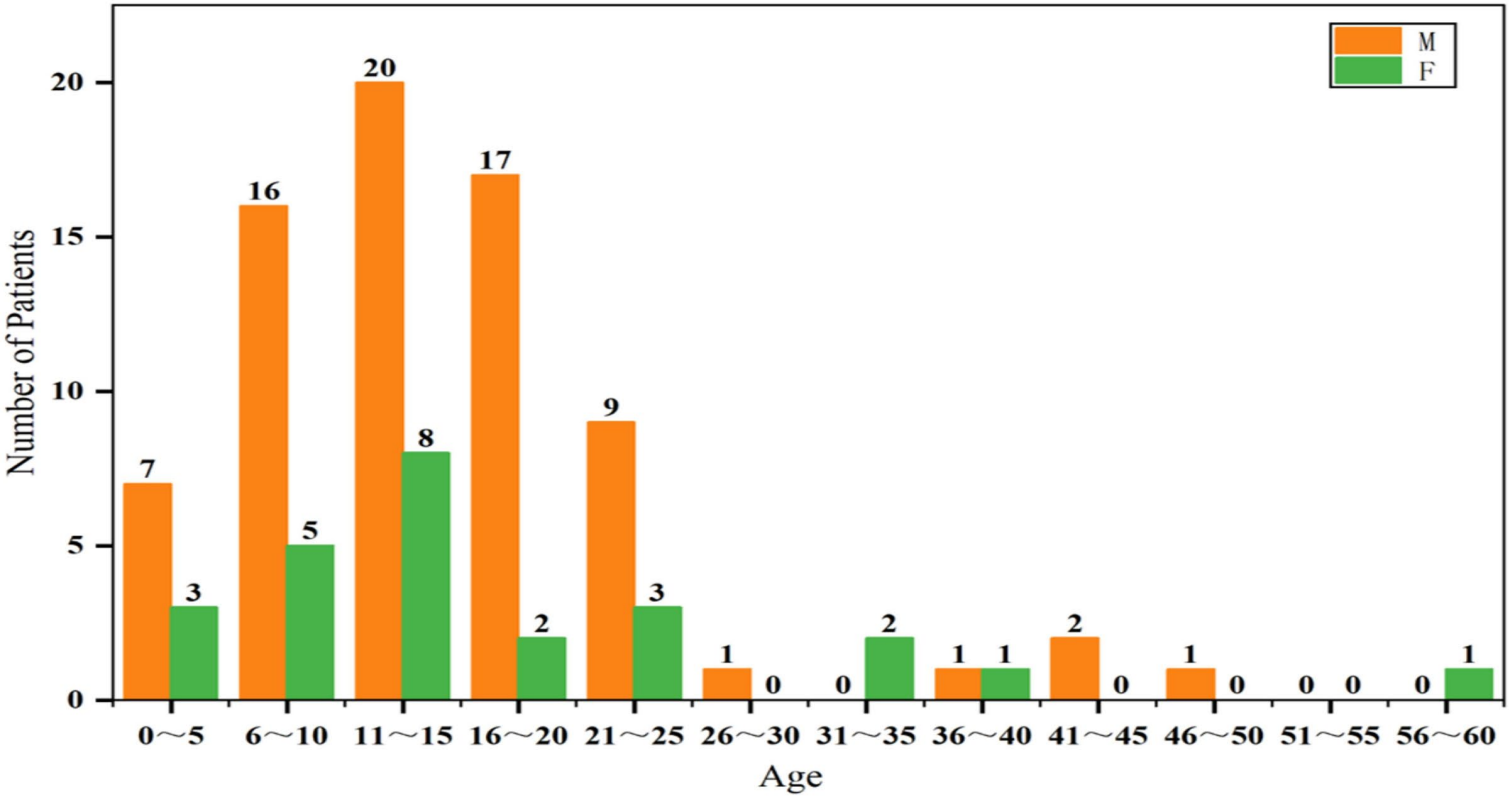
Age and sex distribution of patients with OO.

Of the 99 patients with osteoid osteoma (OO), 74 were male (74.7%) and 25 female (25.3%). Age ranged from 3 to 59 years, with a mean age of 15.41 ± 9.98 years. Onset occurred before the age of 20 years in 80% of patients. Age and sex distribution of patients with OO is shown in Figure 7.

Of the 99 patients, 60 were affected on the right side and 39 on the left. One patient presented with multiple nidus located in the femoral shaft, while 98 had solitary nidus. Including proximal femur (*n* = 42), femoral shaft (*n* = 11), distal femur (*n* = 5), proximal tibia (*n* = 8), tibial shaft (*n* = 10), distal tibia (*n* = 2), proximal humerus (*n* = 2), distal humerus (*n* = 1), talar neck (*n* = 3), distal phalanges (*n* = 3), distal metacarpals (*n* = 2), vertebral appendages (*n* = 3), acetabulum (*n* = 4), scapula (*n* = 1), fibula (*n* = 1), and superior ramus of the pubis (*n* = 1) intra-articular (*n* = 23), extra-articular (*n* = 76).

Among the 99 patients, 89 could be clearly classified: 62 were cortical type, and 27 were endosteal type.

### 4.2 Multimodal imaging findings of OO

The detection rates of bone sclerosis on DR, CT, and MRI were 69.0, 91.9, and 44.4%, respectively; those of nidus were 52.4, 100, and 58.3%, respectively; and combined detection rates of nidus and bone sclerosis were 45.2, 91.9, and 36.1%, respectively. CT showed a detection rate of 89.5% for intranidal calcification, 36.7% for the vascular groove sign, and 52.3% for soft tissue oedema. Among the 36 patients who underwent MRI, 30 (83.3%) had bone marrow edema, 21 (58.3%) had soft tissue edema, and 20 (55.6%) had both bone marrow edema and soft tissue edema. Four (11.1%) had neither. Detection rates of imaging features of OO on DR, CT and MRI is shown in Table 1.

**Table 1:** Detection rates of imaging features of OO on DR, CT, and MRI.

Imaging features	Examination method		
	DR (*n* = 84)	CT (*n* = 86)	MRI (*n* = 36)
Prevalence of bone sclerosis, *n*(%)[95% CI]	58 (69.0) [58.6, 77.7]	79 (91.9) [84.0, 96.2]	16 (44.4) [28.9, 61.0]
Prevalence of nidus, *n*(%)[95% CI]	44 (52.4) [41.9, 62.7]	86 (100.0) [95.7, 100.0]	21 (58.3) [41.4, 73.4]
Prevalence of nidus + sclerosis, *n* (%)[95% CI]	38 (45.2) [35.1, 55.8]	79 (91.9) [84.0, 96.2]	13 (36.1) [21.8, 53.8]
Calcification within the nidus, *n* (%)[95% CI]	14 (16.7) [10.2, 25.8]	77 (89.5) [81.2, 94.4]	20 (55.6) [38.9, 71.1]
Bone marrow oedema, *n*(%)[95% CI]	N/A	N/A	30 (83.3) [67.4, 92.6]
Vascular groove sign, *n*(%)[95% CI]	N/A	23 (26.7) [18.2, 37.1]	N/A
Soft tissue oedema, *n*(%)[95% CI]	N/A	45 (52.3) [41.9, 62.5]	21 (58.3) [41.4, 73.4]

N/A: not available; CI, confidence interval.

### 4.3 Comparison of diagnostic concordance rates for OOs

There was a statistically significant difference in the overall diagnostic concordance rates between DR, CT, and MRI (Fisher’s exact test, *p* < 0.001). Pairwise comparisons (Bonferroni correction) revealed that the overall diagnostic concordance rate for CT was significantly higher than that for DR (*p* < 0.001) and MRI (*p* = 0.004), whereas the difference between DR and MRI was not statistically significant (*p* = 0.605). Comparison of diagnostic concordance for OO among DR, CT and MRI is shown in Table 2. Inter-reader agreement was excellent for nidus detection (*κ* = 0.82, 95%CI: 0.72–0.92), bone sclerosis detection (*κ* = 0.78, 95%CI: 0.67–0.89), and overall diagnostic concordance rate (*κ* = 0.85, 95%CI: 0.76–0.94). For the remaining imaging features (Calcification within the nidus, vascular groove sign, bone marrow edema, and soft tissue edema), inter-reader agreement ranged from 0.75 to 0.89, indicating good to excellent agreement.

**Table 2:** Comparison of diagnostic concordance for OO among DR, CT, and MRI.

Examination method	suggestive diagnosis rate *n*(%)[95% CI]	Definitive diagnosis rate *n*(%)[95% CI]	Overall diagnostic concordance rate *n*(%)[95% CI]
DR (*n* = 84)	25 (29.7) [20.7, 40.4]	44 (52.4) [41.9, 62.7]	69 (82.1) [72.6, 89.0]
CT (*n* = 86)	0 [0.0, 4.3]	86 (100) [95.7, 100.0]	86 (100) [95.7, 100.0]
MRI (*n* = 36)	10 (27.8) [15.6, 44.4]	21 (58.3) [41.4, 73.4]	31 (86.1) [70.5, 94.1]

CI, confidence interval. Overall diagnostic concordance rate = (definitive + suggestive)/total × 100%. Overall comparison among three modalities: Fisher’s exact test, *p* < 0.001. Pairwise comparisons (Bonferroni correction, *α*’ = 0.0167): CT vs DR, *p* < 0.001; CT vs MRI, *p* = 0.004; DR vs MRI, *p* = 0.605.

### 4.4 Presentation of the nidus

Of the 99 patients, 95 had a single nidus, 1 had two nidus, and 3 had not undergone CT or MRI. No definite nidus was identified on DR, resulting in a total of 97 evaluable nidus. The niduses were circular in 17 cases and elliptical in 80 cases: in long bones, 15 circular and 72 were elliptical (elliptical predominance, 82.8%); in flat, short, and irregular bones, 8 were circular and 2 were elliptical (circular predominance, 80.0%). The transverse diameter of the nidus ranged from 0.15 cm to 1.2 cm, with a mean of 0.59 ± 0.19 cm; only three cases had a transverse diameter greater than 1.0 cm. The longitudinal diameter of the nidus ranged from 0.2 cm to 1.7 cm, with a mean of 1.00 ± 0.34 cm; 61.9% were 1 cm or less, and 38.1% were approximately 1 cm.

### 4.5 Characteristics of intra-articular OO

All 23 cases of intra-articular OOs were located in the hip joint, comprising 11 on the left and 13 on the right; 20 were in the femoral neck and 3 in the acetabulum. All cases were associated with joint effusion and synovitis.

## 5 Discussion

### 5.1 Overview of OOs

OOs may originate in the subperiosteal region and subsequently manifest as intramedullary lesions. The site of origin appears to be associated with continuous bone remodelling resulting from subperiosteal deposition and intramedullary erosion (15). Several location-based classification systems for OOs have been proposed in the literature, including three types: subperiosteal, cortical, and cancellous (medullary); or four types: subperiosteal, cortical, endocortical, and intramedullary, with the cortical type being the most common (15, 16).

In this study, the location of the nidus could be clearly assessed in 89 patients: in 11 case, the nidus were partially located within the cortical bone; in 52 cases, they were located within the cancellous bone adjacent to the cortical bone. No cases were observed in which the nidus were entirely located within the medullary cavity. In practice, it is difficult to identify the periosteum on thickened cortical bone (17); furthermore, since the subperiosteal type and the cortical type are similar in anatomical location and treatment approach, we have combined the two into the “cortical type.” The “medullary type” reported in previous literature may be a cross-sectional illusion. Upon examining transverse, sagittal, and coronal views, we found that in every position, the nidus was closely associated with the endosteum; therefore, we are more inclined to classify it as the “endosteal type” rather than as a truly independent intramedullary lesion.

Based on the imaging characteristics, pathogenesis, and therapeutic considerations described above, we propose a simplified two-type classification “cortical type” and “endosteal type.” This simplified classification aligns with the pathogenesis model that OO originates subperiosteally and progressively extends toward the medullary cavity (15). It also offers practical value for clinical management: for radiofrequency ablation, distinguishing between cortical and endosteal locations facilitates precise puncture path design and ablation range selection cortical-type lesions require penetration through dense cortical bone to reach the nidus, whereas endosteal-type lesions require full-thickness cortical penetration into the medullary cavity, where intramedullary blood flow may affect heat distribution during ablation (18). For surgical excision, cortical-type lesions require only partial cortical removal or penetration, while endosteal-type lesions necessitate full-thickness cortical opening to access the medullary cavity, with differences in surgical trauma and bone defect repair (6). However, we acknowledge that this classification has not yet been validated against established systems, and 10 of 99 patients (10.1%) could not be classified due to the lack of cross-sectional imaging (CT or MRI). Further studies with larger sample sizes and formal clinical outcome validation are needed to confirm its clinical utility.

### 5.2 Imaging characteristics of nidus

Previous studies (5, 19) have suggested that nidus are predominantly round or oval in shape. In the present study, niduses in long bones were predominantly elliptical (82.8%), whereas those in flat, short and irregular bones were predominantly circular (80.0%). This difference may be related to the geometric morphology and cortical structure of the different bone types. Calcification or ossification within the nidus appeared as punctate, amorphous or annular patterns, consistent with previous literature reports (4); these variations may reflect different pathological stages of ossification, ranging from low-mineralised osteoid to more mature woven bone. This study found that approximately 10% of nidus showed no calcification on CT scans, whereas previous literature rarely reported statistics on the incidence of calcification within nidus. Nidus exhibit slightly increased signal intensity on both T1-weighted and T2-weighted sequences, which is associated with the nidus matrix being composed of highly vascularised fibrous connective tissue; furthermore, MRI enhancement revealed that, apart from the central area of calcification or ossification which showed no enhancement, the nidus demonstrated marked enhancement, suggesting a rich blood supply (20).

### 5.3 Imaging techniques

Although DR examinations are inexpensive and widely used, they have a low detection rate for intra-articular and spinal lesions, as well as for small nidus and/or those with marked cortical thickening; their detection rate for calcifications within nidus is even lower. Jordan et al. (21) conducted a meta-analysis of 223 patients with confirmed OOs and found that the detection rate on plain radiographs was only 66%. MRI is highly sensitive to bone marrow oedema and soft tissue reactions; however, bone marrow oedema can easily mask small nidus that also exhibit high signal intensity, particularly those without significant central calcification. It has been reported that when MRI is used as the primary imaging modality, the misdiagnosis rate can reach 35% (21–23). In recent years, studies using dynamic contrast-enhanced MRI have revealed vascular-like high signal intensity within nidus during the early arterial phase, significantly improving the detection rate of nidus on MRI (24–26). CT offers high spatial resolution and can clearly display the morphology of small nidus, internal calcifications and surrounding cortical reactions; it is currently the preferred imaging modality for detecting and localising OO nidus (27). In this study, the detection rates of nidus and calcifications on CT were 100 and 89.5% respectively, significantly higher than those of DR and MRI. Within the proliferating bone tissue of OO, fine curved or serpentine low-density grooves, known as the vascular groove sign, are commonly observed (28). Liu et al. (20) reviewed CT images from 42 patients with pathologically confirmed OOs; two radiologists assessed the presence of the vascular groove sign in these images and found that the sign demonstrated moderate sensitivity and high specificity in distinguishing OOs from other bone lesions. In this study, the detection rate of the vascular groove sign was only 26.7%, which is lower than that reported in previous literature. In terms of diagnostic concordance, the overall concordance rate for CT diagnosis was 100%, significantly higher than the 82.1 and 86.1% rates for DR, making it the optimal imaging modality.

### 5.4 Characteristics of intra-articular OOs

Intra-articular OOs are often easily confused with other joint lesions, presenting significant challenges in both clinical and radiological diagnosis (29–31). A retrospective study comparing intra-articular and extra-articular OOs found that the average time to diagnosis was 26 months for intra-articular OOs, compared with 9 months for extra-articular OOs (32). Due to the absence of a periosteum, in some cases, intra-articular OOs may appear on imaging as merely round or oval lucent areas, without evidence of bone proliferation. Other characteristic MRI features include synovitis, joint effusion and periarticular soft tissue oedema. In this study, all 23 cases of intra-articular OOs were associated with synovitis and joint effusion. The association of intra-articular OOs with synovitis and joint effusion may be related to the production of inflammatory mediators, such as prostaglandins, by the nidus, which directly stimulate the adjacent synovium, leading to synovial congestion, inflammatory cell infiltration and increased joint effusion, thereby causing joint effusion and synovitis (33). Clinically, intra-articular OOs primarily manifest as joint tenderness, stiffness, limited range of motion and pain; typical nocturnal pain is usually not prominent (29). These symptoms are also present in synovitis or infectious arthritis caused by other aetiologies, and the condition is therefore easily misdiagnosed as simple synovitis. Consequently, in patients presenting with unexplained joint effusion and persistent joint pain, a careful search for occult nidus around or within the joint should be undertaken, combined with precise localisation via CT, to differentiate the condition from infectious lesions and other neoplastic conditions.

## 6 Limitations of the study

Several limitations of this study should be acknowledged. First, the modality subgroups were unequal and overlapping (84 DR, 86 CT, 36 MRI), with only 35 patients undergoing all three modalities, which may introduce heterogeneity. Second, the limited MRI sample size (*n* = 36) reduces statistical power for MRI-related comparisons. Third, although inter-reader agreement was good, the evaluations were performed with knowledge of the final diagnosis, which may introduce interpretation bias. Fourth, the perfect concordance rate for CT is susceptible to verification bias, as histopathological confirmation likely favored CT-detectable lesions. Fifth, this was a retrospective, two-center study, and the findings require validation in prospective, multi-center cohorts.

## 7 Conclusion

CT is significantly superior to DR and MRI in visualising the nidus of OO, as well as the surrounding bone sclerosis and calcifications within the nests; with an overall diagnostic concordance rate of 100%, it is the optimal imaging modality. DR is cost-effective and widely used, playing a vital role in initial screening; although MRI has limited ability to directly visualise the nidus, it offers unique advantages in demonstrating bone marrow and soft tissue oedema, and is particularly valuable for assessing the activity of intra-articular lesions. Intra-articular OOs are often associated with marked synovitis and joint effusion, which warrants clinical attention.

## Data Availability

The original contributions presented in the study are included in the article/supplementary material, further inquiries can be directed to the corresponding author.
